# Persistent Pyelonephritis and Renal Abscess due to *Mycoplasma hominis* in an Immunocompromised Patient: A Case Report

**DOI:** 10.1155/crdi/8602744

**Published:** 2026-06-27

**Authors:** Lydia M. Hill Almeida, Dujinthan Jayabalan, Kelvin Chan, Chan Y. Cheah, Thomas Gliddon

**Affiliations:** ^1^ Department of Haematology, Sir Charles Gairdner Hospital, Nedlands, Western Australia, Australia, scgh.health.wa.gov.au; ^2^ Medical School, The University of Western Australia, Perth, Western Australia, Australia, uwa.edu.au; ^3^ Linear Clinical Research, Nedlands, Western Australia, Australia; ^4^ Department of Infectious Diseases, Sir Charles Gairdner Hospital, Nedlands, Western Australia, Australia, scgh.health.wa.gov.au

**Keywords:** atypical pathogen, case report, culture-negative infection, opportunistic infection, urosepsis

## Abstract

**Background:**

*Mycoplasma hominis* is a fastidious, cell wall‐deficient bacterium commonly colonising the lower genitourinary tract. While usually commensal, it can cause opportunistic infections in immunocompromised patients. As it can be difficult to culture on standard mediums and is resistant to many first‐line antimicrobials, diagnosis and treatment are often delayed.

**Case Presentation:**

We report the case of a 31‐year‐old woman with Stage IVA extranodal marginal zone lymphoma previously treated with bendamustine‐rituximab, who presented with fever, suprapubic pain and haematuria in the setting of neutropenia. She developed acute kidney injury and was initially treated for neutropenic sepsis. Computed tomography revealed bilateral hydroureteronephrosis and, subsequently, a right renal lesion concerning for abscess. Despite broad‐spectrum empirical therapy, she remained febrile with persistently elevated inflammatory markers. Blood cultures grew *Streptococcus mitis* in a single bottle, later considered a contaminant. Routine urine cultures were initially negative; *M. hominis* was identified only after prolonged incubation and directed testing on a urine culture collected on Day 5 of admission. On Day 10 of admission, urine culture identified *Mycoplasma hominis*. Directed therapy with intravenous clindamycin, doxycycline and levofloxacin led to clinical improvement. She was discharged after 17 days with a combination of oral doxycycline and levofloxacin. She was readmitted shortly afterwards with symptom recurrence and progression of renal abscesses but responded to re‐initiation of clindamycin. At outpatient follow‐up, she had transitioned to levofloxacin monotherapy with symptomatic improvement, radiological reduction of abscesses, downtrending inflammatory markers and recovery of renal function.

**Conclusion:**

This case highlights *M. hominis* as a rare but important cause of upper urinary tract infection and renal abscess in immunocompromised hosts. Clinicians should suspect atypical pathogens in culture‐negative urosepsis unresponsive to empirical antibiotics. Specialised diagnostics are essential for accurate detection, and timely targeted therapy can achieve favourable outcomes even when surgical drainage is not feasible.

## 1. Introduction

*Mycoplasma hominis* is a member of the Mollicutes, the smallest self‐replicating prokaryotes [1]. These organisms lack a cell wall, explaining both their dependence on host cells for biosynthesis and their intrinsic resistance to β‐lactam antibiotics [1]. Although commonly regarded as commensals of the lower genitourinary tract, particularly in sexually active individuals [2], *M. hominis* has been implicated in urogenital tract infections such as bacterial vaginosis, pelvic inflammatory disease, and urinary tract infections [3, 4], as well as rarer extragenital infections including pyelonephritis [5] and perinephric abscesses [6, 7], amongst other infections [8–11].

Unlike other Mycoplasma species, *M. hominis* is capable of limited growth on routine blood agar, forming minute colonies after prolonged incubation which are frequently overlooked. When clinical suspicion exists, laboratories should be informed so that extended incubation and specialised media can be used. This diagnostic difficulty leads to frequent underdiagnosis and delayed treatment. When identified, management is further complicated by intrinsic resistance to β‐lactams, sulfonamides, trimethoprim and rifampin, leaving tetracyclines, lincosamides, macrolides and fluoroquinolones as the principal active agents [1, 12]. Emerging resistance, particularly to tetracyclines [13] and fluoroquinolones [14], has also been reported.

Reports of *M. hominis* pyelonephritis and renal abscess are uncommon, and most involve immunocompromised hosts such as transplant recipients [6, 7]. Here, we describe a case of persistent pyelonephritis and renal abscess due to *M. hominis* in a neutropenic patient with a history of lymphoma, highlighting the importance of considering atypical urogenital pathogens when routine cultures remain negative. This case is notable for describing persistent upper urinary tract infection and renal abscess in a non‐transplant lymphoma patient managed successfully without surgical drainage. Key teaching points include the diagnostic difficulty posed by this fastidious organism, the need for clinical suspicion in culture‐negative urosepsis, and the role of targeted antimicrobial therapy in achieving favourable outcomes.

## 2. Case Presentation

A 31‐year‐old female presented with a 3‐day history of suprapubic pain, macroscopic haematuria and fever. She had a background of Stage IVA extranodal marginal zone lymphoma treated with six cycles of bendamustine‐rituximab without maintenance rituximab, achieving a complete metabolic response 6 months prior to presentation. Her prior medical history included recurrent haemorrhagic cystitis with negative standard urine cultures and biopsy‐proven interstitial cystitis at a different hospital, which had been treated empirically with amoxicillin‐clavulanic acid with partial symptom resolution. Her only other significant history was major depression with atypical features.

On admission, she was febrile to 41.0°C, tachycardic and neutropenic (neutrophils 0.35 × 10^9^/L). C‐Reactive protein (CRP) was markedly elevated at 325 mg/L. She had an acute kidney injury, with a creatinine of 151 μmol/L (baseline 69) and eGFR 39 mL/min/1.73 m^2^ (previously > −90). Lymphocyte subset analysis demonstrated reduced absolute counts across all subsets except CD16/56+ NK cells, with a CD4+ T‐cell count of 65 cells/μL. Her serum immunoglobulin levels were within normal limits (IgG 11.6 g/L, IgA 2.49 g/L, IgM 0.99 g/L). Initial management included intravenous piperacillin‐tazobactam 4.5 g every 6 hours for neutropenic sepsis, fluconazole 400 mg once daily and a single subcutaneous dose of 300 μg granulocyte colony‐stimulating factor.

**FIGURE 1:**
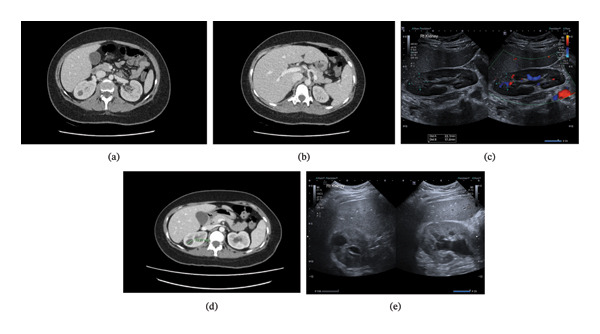
Imaging evolution of renal abscess. (a) Abdominal computed tomography imaging (Day 8 of admission) showing a 15 mm low‐attenuation lesion at the right upper renal pole. (b) Abdominal computed tomography imaging (Day 8 of admission) showing ill‐defined low‐attenuation changes at the left upper renal pole. (c) Ultrasound imaging of the urinary tract (Day 8 of admission) showing an oedematous right kidney with an ill‐defined focus of increased cortical echogenicity in the upper pole measuring 22 × 17 mm. (d) Abdominal computed tomography (Day 19 since initial admission) showing interval increase in size of right upper pole hypoattenuating lesion, measuring maximum of 19 mm. (e) Abdominal ultrasound imaging of right kidney (Day 24 since initial admission) showing a solitary hypoechoic, smooth margins, noncalcified and right upper pole lesion measuring 21 × 16 mm with moderate hydronephrosis.

Computed tomography of the kidneys, ureters and bladder (CT KUB) demonstrated bilateral hydroureteronephrosis to the level of the vesicoureteric junction, without obstructing calculi or masses Figure 1. Mild bladder distension with perivesical fat stranding was noted. An indwelling catheter was placed for bladder decompression, and oxybutynin (5 mg three times daily) was commenced.

Urine microscopy demonstrated marked pyuria and haematuria, although culture initially yielded only scant mixed growth. Admission blood cultures grew *Streptococcus mitis* in a single aerobic bottle, prompting commencement of intravenous vancomycin; this was later considered a contaminant, as all subsequent cultures were sterile. Comprehensive infectious screening including Mycoplasma genitalium polymerase chain reaction (PCR) in both plasma and urine, respiratory virus PCR (including Influenza A, Influenza B, human metapneumovirus, parainfluenza virus and respiratory syncytial virus), Hepatitis A Virus serology, Hepatitis B Virus serology, enteric PCR and stool ova, cysts and parasites was negative. Other potential causes of culture‐negative haemorrhagic cystitis, including BK virus, JC virus, adenovirus and atypical mycobacteria, were excluded by targeted PCR and culture testing. Transthoracic echocardiography revealed no features of infective endocarditis. Despite broad‐spectrum antimicrobial therapy, including trials of ceftriaxone and meropenem, the patient remained intermittently febrile with persistently elevated inflammatory markers.

Repeat imaging on Day 8 of admission revealed a 15 mm low‐attenuation lesion in the right upper pole communicating with the renal pelvis, as well as ill‐defined changes in the left kidney consistent with focal nephronia (Figure 1a,b). Moderate bilateral hydronephrosis persisted. Ultrasound confirmed bilateral oedematous kidneys with focal nephronia but no drainable collection (Figure 1c).

On Day 10 of admission, the urine culture collected on Day 5 grew *Mycoplasma hominis* (> 100 white blood cell count, > 100 red blood cell count, no nitrites). This was considered the likely pathogen, and intravenous clindamycin (600 mg three times daily) was initiated, with the subsequent addition of levofloxacin (750 mg once daily). All other antimicrobials were ceased. Neutropenia resolved on Day 11. The ammonia level was not elevated, with a plasma level of 10 µmol/L.

Despite broad‐spectrum antibiotics (piperacillin‐tazobactam, ceftriaxone, meropenem and vancomycin), the patient had persistent fevers until the initiation of clindamycin directed against *M. hominis*. She was transitioned to oral doxycycline and levofloxacin as inflammatory markers improved (CRP decreased from a peak of 325 mg/L to 217 mg/L).

She was discharged after a 17‐day admission with ongoing catheter drainage, oral doxycycline (100 mg twice daily) and levofloxacin (750 mg daily), planned to continue for four weeks.

Two days later, she re‐presented with worsening abdominal pain and rising CRP (258 mg/L). CT abdomen demonstrated progression of bilateral pyelonephritis with an enlarging right renal abscess and multiple new hypoattenuating renal lesions, and bilateral hydroureteronephrosis with urothelial thickening and enhancement (Figure 1d). Ultrasound again showed no drainable collections (Figure 1e). All repeat blood and urine cultures were sterile.

She was recommenced on intravenous clindamycin, alongside oral levofloxacin and doxycycline, with clinical improvement and resolution of fevers. At discharge, CRP had decreased to 127 mg/L. Renal function remained impaired but stable (creatinine 176 μmol/L, eGFR 33). She was discharged with home intravenous clindamycin via a peripherally inserted central catheter, in combination with oral doxycycline and levofloxacin, with a planned four to eight weeks of therapy.

At outpatient follow‐up, she remained catheter‐dependent but was clinically improving on her antibiotic regimen. Interval imaging showed a reduction in the size of the renal abscesses; however, new omental and peritoneal soft‐tissue infiltrates were noted extending through the epigastrium, splenic hilum and left paracolic gutter, with inflammatory stranding abutting the tail of the pancreas. In the context of left upper quadrant pain and an elevated lipase (3920 U/L), this initially prompted a clinical diagnosis of pancreatitis and led to the cessation of doxycycline due to concern for doxycycline‐induced pancreatitis. Although initially treated as pancreatitis, the picture was later re‐interpreted as immune reconstitution inflammatory syndrome (IRIS) in the context of CD4 recovery. During this period, she also developed a mild truncal pruritic rash, thought to represent a delayed antimicrobial‐related reaction to clindamycin or possible doxycycline‐associated photosensitivity; given this concern, doxycycline and clindamycin were discontinued and she was transitioned to levofloxacin monotherapy. The rash resolved following drug cessation. She was subsequently ceased all antimicrobials following 8 weeks of therapy. At the time of reporting, she remained clinically well with improving symptoms, downtrending inflammatory markers (CRP 7 mg/L) and normalised renal function. Follow‐up CT imaging demonstrated complete resolution of the previously noted peritoneal and omental infiltrates. Patient’s clinical progress is summarised in Table 1.

**TABLE 1:** Clinical timeline from presentation to follow‐up.

Day	Event
0	Admission; fever, haematuria, neutropenia, AKI; empirical broad spectrum antibiotics commenced
8	CT/USS: right renal abscess and focal nephronia; no drainable collection
10	*M. hominis* identified on urine culture; IV clindamycin and levofloxacin commenced; all prior antibiotics ceased
11	Neutropenia resolved; clinical improvement; step‐down to oral doxycycline and levofloxacin
17	Discharged on oral doxycycline and levofloxacin
19	Readmission; abscess progression on CT; IV clindamycin recommenced, oral doxycycline and levofloxacin continued
26	Discharged on IV clindamycin via PICC, oral doxycycline and levofloxacin
∼42	Clindamycin and doxycycline ceased due to rash, continued levofloxacin monotherapy
∼56	All antibiotics ceased; CRP 7 mg/L; CT confirmed complete resolution; renal function normalised

*Note:* IV, intravenous; USS, ultrasound.

Abbreviations: AKI, acute kidney injury; CRP, C‐reactive protein; CT, computed tomography; PICC, peripherally inserted central catheter.

## 3. Discussion

We describe a rare case of *Mycoplasma hominis* causing persistent pyelonephritis with renal abscess formation in a neutropenic patient with a history of marginal zone lymphoma. This case reinforces the diagnostic challenges and associated delays in appropriate treatment associated with this organism and hence the need for clinicians to maintain a high degree of suspicion for atypical organisms, particularly in immunocompromised patients.

*M. hominis* is a fastidious, slow‐growing organism that lacks a cell wall and does not produce nitrites on urine dipstick testing [1, 4]. These features make it difficult to detect with routine culture methods or gram staining. *M. hominis* is unique among Mycoplasma species in that it can occasionally grow on standard blood agar after prolonged incubation, forming pinpoint colonies that are easily overlooked. In this case, *M. hominis* was identified after several days of incubation on routine blood agar, where its fastidious growth was noted and subsequently confirmed as the causative pathogen. In many previously published reports, however, *M. hominis* was detected only when routine cultures remained negative and specialised media or molecular methods were used [15, 16]. Immunosuppression further complicates recognition: neutropenia, lymphopenia, hypogammaglobulinemia and prior antibiotic exposure can blunt typical clinical and laboratory findings. Cases of *M. hominis* pyelonephritis or abscess, particularly in transplant recipients, have similarly been diagnosed only after failure of empirical β‐lactam therapy and use of molecular or targeted culture techniques [15–17]. Metagenomic next‐generation sequencing (mNGS) is an emerging diagnostic modality increasingly used in immunocompromised patients with suspected invasive infections. It can rapidly detect M. hominis from plasma, even in cases where conventional cultures fail, and should be considered where available when atypical pathogens are suspected and standard testing is unrevealing.

Therapy is challenging due to intrinsic resistance to β‐lactams, sulphonamides, trimethoprim, rifampin and often macrolides. In this case, broad‐spectrum agents including vancomycin, ceftriaxone and meropenem were ineffective. Fever and inflammation resolved only after initiation of clindamycin, later combined with doxycycline and levofloxacin. The temporal resolution of fever and inflammatory markers after starting targeted antibiotic therapy supports *M. hominis* as the causative pathogen. The literature supports tetracyclines and fluoroquinolones as first‐line agents where susceptible, with clindamycin also effective in many cases [4, 18]. Successful use of doxycycline, moxifloxacin or combination regimens has been reported in prior cases of *M. hominis* pyelonephritis and renal abscesses [5, 6]. The duration of therapy remains uncertain; in this patient, prolonged treatment and serial imaging were required as drainage was not feasible.

Standardised methods for antimicrobial susceptibility testing of *M. hominis* have been established by the Clinical and Laboratory Standards Institute (CLSI), although their clinical validation remains limited. Resistance patterns vary substantially between regions, with high rates of macrolide resistance and variable susceptibility to tetracyclines and fluoroquinolones, whereas clindamycin and pristinamycin typically retain activity [12]. In this case, antimicrobial susceptibility testing was not yet performed on the stored isolate but may be pursued if relapse occurs, particularly given the patient’s probable clindamycin‐induced rash. As supported by previous case reports, prolonged combination therapy is often required in severe infection. Clindamycin appeared to be the key active agent in this case, consistent with its generally low minimum inhibitory concentrations and lower rates of resistance [12, 19].

Cases of *M. hominis* extragenital and disseminated infection have been previously described in hypogammaglobulinemic patients, most often associated with rituximab‐induced B‐cell depletion [20–22]. Although rare, these reports demonstrate the opportunistic potential of *M. hominis* in immunocompromised hosts. The organism has also been implicated in hyperammonaemia syndrome, typically in donor‐derived infections within the transplant cohort. This patient’s immunocompromised state following bendamustine‐rituximab, together with neutropenia, retention‐associated stasis, prior haemorrhagic cystitis and repeated antibiotic exposure, likely predisposed to *M. hominis* infection. The patient’s improvement coincided with the recovery of CD4 count from 65 to 180 cells/μL, suggesting immune reconstitution may have aided resolution. Chronic colonisation of the lower urinary tract with intermittent invasion under immune suppression may explain the history of recurrent “culture‐negative” haemorrhagic cystitis, which in retrospect may have been attributable to *M. hominis*.

This case highlights the need to consider *M. hominis* in immunocompromised patients with suspected urinary tract infection when routine cultures are negative and empirical broad‐spectrum antibiotics fail. Diagnosis often requires clinician‐directed requests for specialised culture media, prolonged incubation, and dedicated PCR. Where available, antimicrobial susceptibility testing should guide treatment selection, given increasing resistance to tetracyclines and fluoroquinolones. Management requires agents with reliable activity, such as clindamycin, doxycycline and fluoroquinolones; prolonged therapy; and close monitoring with imaging to confirm resolution. In selected cases with small abscesses not amenable to drainage, conservative medical management may be sufficient. Attention should also be paid to antibiotic toxicities and drug interactions in immunocompromised patients.

It remains unclear whether earlier initiation of *M. hominis*‐directed therapy could have prevented abscess formation in this case or whether prior episodes of haemorrhagic cystitis represented unrecognised *M. hominis* infection. Long‐term outcomes, including renal function, recurrence risk and bladder health, require ongoing follow‐up.

## 4. Conclusion

*M. hominis* should be recognised as a potential cause of severe upper urinary tract infection and renal abscess in immunocompromised hosts. Because routine diagnostic methods often fail, clinicians should maintain suspicion for atypical pathogens in immunocompromised patients with culture‐negative urinary sepsis and consider specialised testing to ensure timely diagnosis and treatment. Targeted antimicrobial therapy can lead to clinical improvement even when surgical drainage is not feasible. Earlier clinical suspicion and directed testing may shorten the time to appropriate therapy and reduce unnecessary broad‐spectrum antibiotic exposure.

## Author Contributions

L.M.H.A.: conceptualisation, data curation, writing–original draft and project administration. D.J.: writing–review and editing. K.C.: writing–review and editing. C.Y.C.: supervision and writing–review and editing. T.G.: supervision and writing–review and editing.

## Funding

No funding was received for this research.

Open access publishing was facilitated by The University of Western Australia, as part of the Wiley‐The University of Western Australia agreement via the Council of Australasian University Librarians.

## Ethics Statement

This study was conducted in accordance with the Declaration of Helsinki. Ethical approval to undertake this study was obtained from the Sir Charles Gairdner Osbourne Park Hospital Care Group Quality Improvement Committee.

## Consent

Written informed consent was obtained from the patient for publication of this case report and accompanying images.

## Conflicts of Interest

The authors declare no conflicts of interest.

## Data Availability

The data that support the findings of this study are available on request from the corresponding author. The data are not publicly available due to privacy or ethical restrictions.
